# The effect of reward-induced arousal on the success and precision of episodic memory retrieval

**DOI:** 10.1038/s41598-024-52486-6

**Published:** 2024-01-24

**Authors:** Beth Lloyd, Sander Nieuwenhuis

**Affiliations:** 1 Institute of Psychology, Leiden University, Wassenaarseweg 52, 2333 AK Leiden, The Netherlands; 2 Leiden Institute for Brain and Cognition, Leiden University, Leiden, The Netherlands

**Keywords:** Psychology, Human behaviour

## Abstract

Moment-to-moment fluctuations in arousal can have large effects on learning and memory. For example, when neutral items are predictive of a later reward, they are often remembered better than neutral items without a reward association. This reward anticipation manipulation is thought to induce a heightened state of arousal, resulting in stronger encoding. It is unclear, however, whether these arousal-induced effects on encoding are ‘all-or-none’, or whether encoding precision varies from trial to trial with degree of arousal. Here, we examined whether trial-to-trial variability in reward-related pupil-linked arousal might correspond to variability in participants’ long-term memory encoding precision. We tested this using a location memory paradigm in which half of the to-be-encoded neutral items were linked to later monetary reward, while the other half had no reward association. After the encoding phase, we measured immediate item location memory on a continuous scale, allowing us to assess both memory success and memory precision. We found that pre-item baseline pupil size and pupil size during item encoding were not related to subsequent memory performance. In contrast, the anticipation of instrumental reward increased pupil size, and a smaller anticipatory increase in pupil size was linked to greater subsequent memory success but not memory precision.

## Introduction

Encoding and retaining valuable information in long-term memory is a highly adaptive process as it allows us to shape our future behavior with improved choices and actions^1^. As such, the potential relevance of an event or our motivational state can modulate the strength of episodic memory formation. Indeed, studies have shown that motivational states associated with anticipating reward (e.g., money, points, calories) improve the accuracy of episodic memories. For example, items that signal opportunities for future rewards are often better remembered than neutral items that do not have any reward association^2,3^. This memory enhancement occurs even when the to-be-remembered items are unrelated to the reward, but presented after a reward-signaling cue and during the anticipatory phase^4,5^. Also, similar effects have been found during incidental memory formation^2,3,6–10^ and intentional memory formation^4,5,11–13^, suggesting that reward-related memory effects are not limited to specific types of encoding.

Emerging evidence suggests that reward promotes episodic memory formation through interactions between the hippocampus and the mesolimbic dopamine system^1,14–16^. Specifically, dopamine release from the substantia nigra/ventral tegmental area (SN/VTA) modulates learning of salient information by enhancing the maintenance of long-term potentiation. In human fMRI studies, co-activation of the SN/VTA and hippocampus during reward anticipation has been found to support reward-related memory benefits^2,4,13^. Recent evidence from animal studies also points to the locus coeruleus (LC) as playing a key role in dopaminergic memory consolidation^17–19^. Despite growing evidence that reward anticipation strengthens the encoding of information, it is unclear whether reward-based memory enhancement and underlying changes in activity of these ascending arousal system nuclei affect only the probability of successfully retrieving information from memory, or also changes in the precision (i.e., quality, fidelity) of the retrieved memory features.

Until recently, long-term memory has typically been studied using categorical measures, for example, by asking participants to indicate whether an item has been seen before (‘old’) or not (‘new’). These methods, however, tend towards characterising episodic recollection as an ‘all-or-none’ process by offering only binary distinctions between successful and unsuccessful memory retrieval. Instead, the recollection of studied items likely operates in a more nuanced ‘some-or-none’ manner, where the precision of the successfully retrieved information is graded^20–22^. In line with the notion that the precision of memory representations can differ, a number of studies have used continuous measures of retrieval performance to gather a more detailed understanding of episodic memory retrieval. Indeed, employing these types of tasks has revealed that retrieval success and retrieval precision are distinct aspects of long-term memory^20,23,24^. Here, we examined whether reward anticipation and corresponding changes in pupil-linked arousal would modulate the precision with which memory representations are formed, much like catecholamine levels and pupil-linked arousal can influence the precision of perceptual representations^25,26^.

To address this question, we examined the relationship between trial-to-trial variability in reward-related arousal and encoding precision using a location memory paradigm containing a monetary incentive delay task component^2,27^. We aimed to distinguish between the probability of successfully retrieving information from memory and the precision of the retrieved memory representation, using Zhang and Luck’s^28^ mixture model. Participants encoded items of which the location varied in a circular space. At test, participants were asked to move the item to the studied location in the same circular space, allowing for sensitive assessment of retrieval performance. We manipulated reward anticipation by presenting participants with neutral items that were either associated with a later reward or had no reward association. To capture moment-to-moment changes in reward-related arousal, we measured pupil size as an indirect measure of activity in the SN/VTA and LC^29–31^. We hypothesized that rewarding items would be associated with increased arousal, as indicated by larger pupil size, compared to neutral items, and that higher reward-related arousal during encoding would be associated with better memory success and higher encoding precision.

## Results

**Figure 1:**
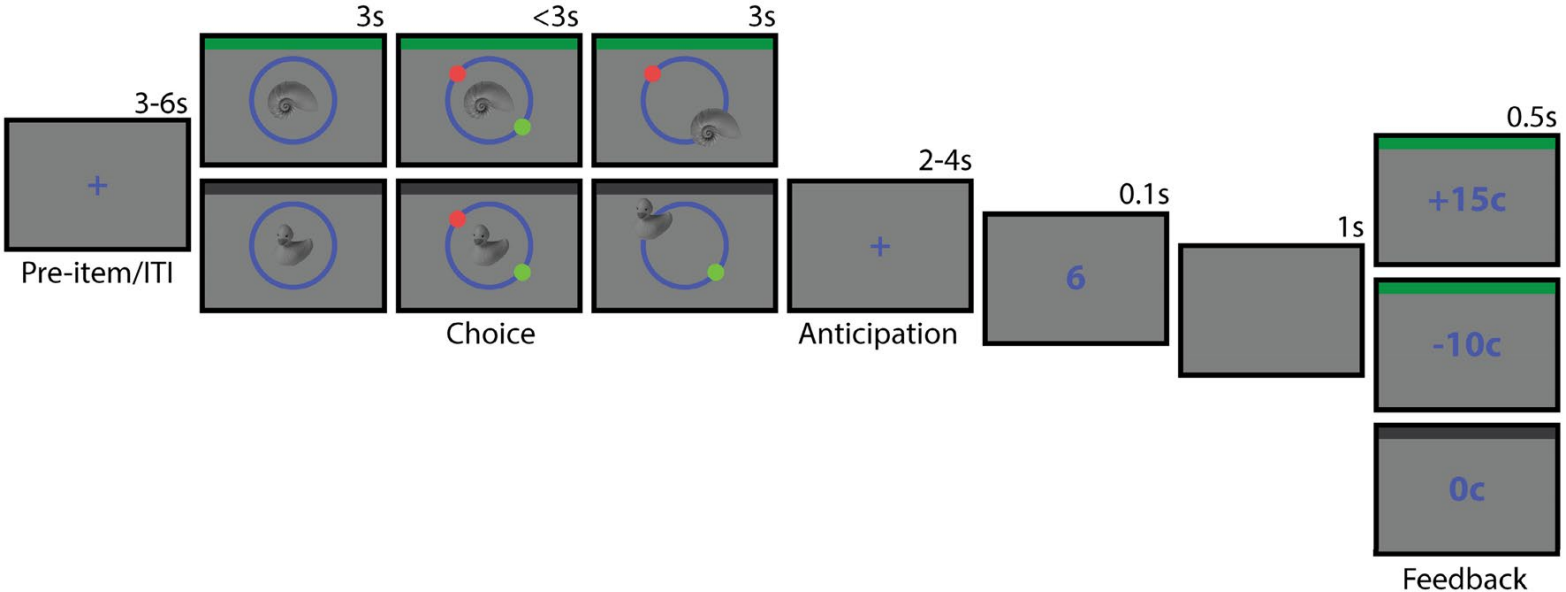
Schematic overview of the study phase. Green top border indicates reward trials (in this example, natural items), grey top border indicates the neutral trials (in this example, man-made items; borders used for illustration purposes only). All colours were equal in luminance to the background colour.

We examined whether trial-to-trial variability in reward-related arousal might correspond to variability in long-term memory encoding. Participants were asked to classify pictures of man-made and natural objects of which one category signaled a potential reward in a subsequent number classification task while the other category did not (Fig. 1). Participants were also asked to encode the location of each item in circular space. After a brief distractor task, participants viewed the previously presented items and were instructed to recall the location associated with each item during the encoding phase. This location memory paradigm, in combination with a model-based analysis of recall errors, allowed us to measure episodic memory precision on a continuous scale (remembered location–encoded location). Therefore, we were able to interrogate memory variability both in terms of the probability of successful retrieval and the precision of item location. We first assessed whether the reward manipulation successfully induced a reward-related arousal response in our participants. To do this, we carried out a number of manipulation checks on behavior and pupil size during item encoding. Next, we evaluated behavioral performance in the memory test, to assess whether rewarding items were remembered better than neutral items. Finally, we explored whether pupil size during item encoding was linked to either memory success or the precision of the recalled location.

### Clear reward anticipation response during task

#### Behavioural data

**Figure 2:**
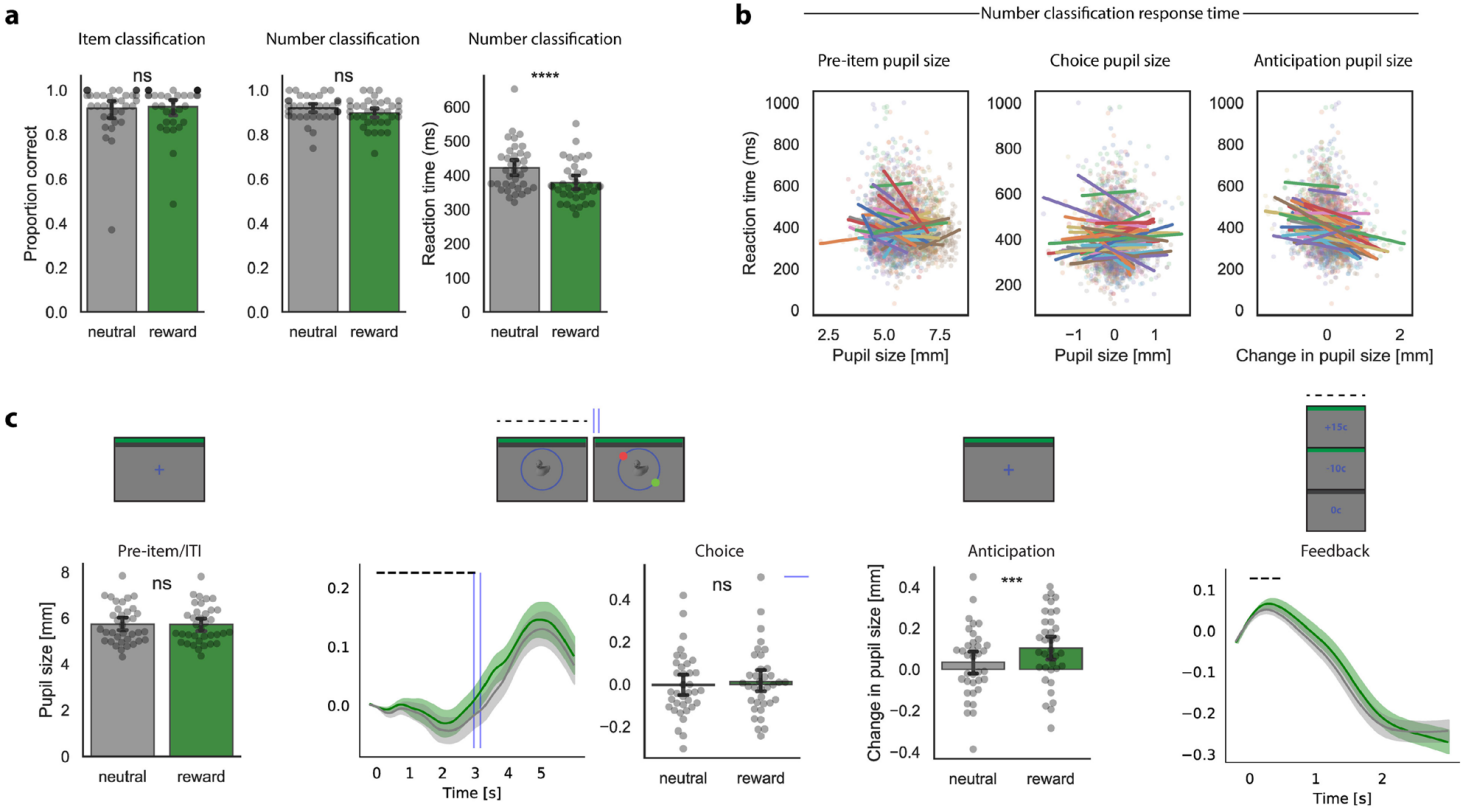
Reward anticipation manipulation during the study phase. (**a**) Bar plots depicting the behavioral indices of the study phase. The proportion of correct responses for item classification (left) and number classification (middle) did not differ between conditions (*p*s > .10). Reaction time on the number classification question (right) was faster for reward trials than neutral trials (*p* < 0.001). (**b**) Reaction time was shorter when the increase in pupil size during the anticipatory phase was larger (right; *p* < 0.001). Pre-item pupil size and pupil size during item classification (choice; middle) did not relate to reaction time on the number classification question (*p*s > 0.07). Regression lines are colored by participant. Points refer to individual trials colored by participant. (**c**) Pupil size for reward and neutral trials during each event of the study phase. The increase in pupil size during the anticipation period was significantly larger on reward trials (*p* < 0.001). Grey points refer to participant means per condition; bars depict condition means; error bars depict 95% confidence intervals at the participant level; shaded areas refer to mean ± SEM; dashed lines refer to the timing of the on-screen event. *p* < .0001: ****; *p* < .001: ***; *p* > .05: ns.

The study phase was made up of two components, the item classification (‘Do you expect a reward on this trial?’) and the number classification (‘Is the number higher orlower than 5?’). Participants performed well on item classification, correctly classifying 93.0 ± 1.7% of reward trials and 92.0 ± 1.9% of neutral trials with no difference between conditions (*p* = 0.54). The proportion of correct responses for the number classification question did not differ between conditions (reward: 89.9 ± 1.0%; neutral: 92.0 ± 0.9%; *p* = 0.10), but reaction times were significantly shorter on reward trials (378 ± 10 ms) than on neutral trials (422 ± 12 ms; *p* < 0.001; Fig. 2a), suggesting that participants were motivated to receive positive feedback on reward trials. Participants earned a reward on an average of 71 ± 2% of the trials, approximating the target of 70% reward trials.

#### Pupil data

As expected, pre-item pupil size was equal across conditions (reward: 5.7 ± 0.13 mm, neutral: 5.7 ± 0.14; *p* = 0.35, BF_10_ = 0.27). We observed a clear reward-related increase in pupil size during the anticipation period of the trial (end of period–start of period), with reward trials inducing a significantly larger pupil response (0.10 ± 0.03 mm) than neutral trials (0.03 ± 0.03 mm; *p* < 0.001; Fig. 2c). The corresponding Bayes factor for the effect of reward anticipation on pupil size revealed strong evidence in favour of the alternative hypothesis (BF_10_ = 73.5). During the choice part of the trial, however, where participants encoded and classified the item as either a reward-signaling or a neutral item, pupil size did not differ between conditions (reward: 0.01 ± 0.03 mm, neutral:  − 0.01 ± 0.03 mm; *p* = 0.33, BF_10_ = 0.28).

#### Pupil and behaviour

Next, on the trial-level we explored whether pupil size during any of the three events (pre-item, choice, anticipation) was associated with a behavioural reward anticipation response (i.e., reaction time to the number classification question; Fig. 2b). Using a linear mixed effects model with the three pupil events and condition (reward vs. neutral) as predictors of interest, task block as a predictor of no interest, and reaction time as an outcome variable, we found that reaction time on the number classification question was associated with the increase in pupil size during the anticipatory period (*t*(35) = − 4.5, *p* < 0.001; Bayesian model *b* = − 29.9, 95% CI [− 44.92,  − 13.48]), over and beyond the effect of condition (*t*(35) = − 5.5, *p* < 0.001, Bayesian model *b* = − 40.37 95% CI [− 53.34,  − 26]). Specifically, for each 1-mm increase in anticipatory pupil size, reaction time to the target number was estimated to decrease by 30 ms. As seen in Fig. 2b (right), in most participants, shorter reaction times corresponded to a larger change in anticipatory pupil size. In contrast, we found no association with pre-item pupil size or pupil size during item classification *t*s < − 1.9, *p*s > 0.07).

Together, these results suggest that the reward anticipation manipulation successfully induced behavioural changes (i.e., reaction time) and stronger change in anticipatory pupil dilation during the study phase. Next, we explored whether reward anticipation affected the precision of item encoding.

### The change in anticipatory pupil size predicts memory success but not memory precision

#### Behavioural data

**Figure 3:**
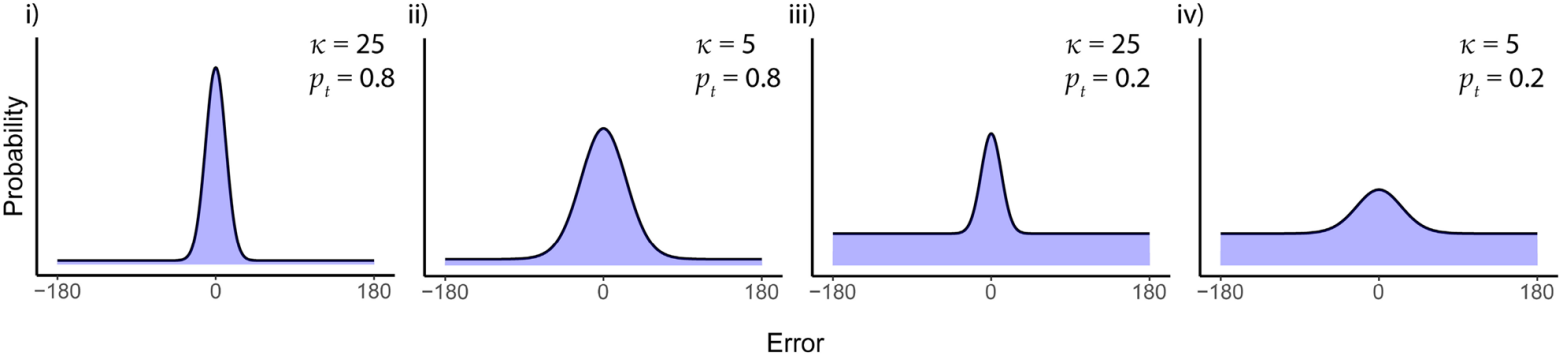
Probability density functions illustrating the different possibilities of memory success and memory precision. We applied the two-component mixture model to simulated data to illustrate different levels of memory success (proportion of responses modelled by a uniform distribution) and memory precision (the concentration of the von Mises distribution). The probability density functions illustrate (**i**) high memory precision, high memory success; (**ii**) low memory precision, high memory success; (**iii**) high memory precision, low memory success; (**iv**) low memory precision, low memory success.

**Figure 4:**
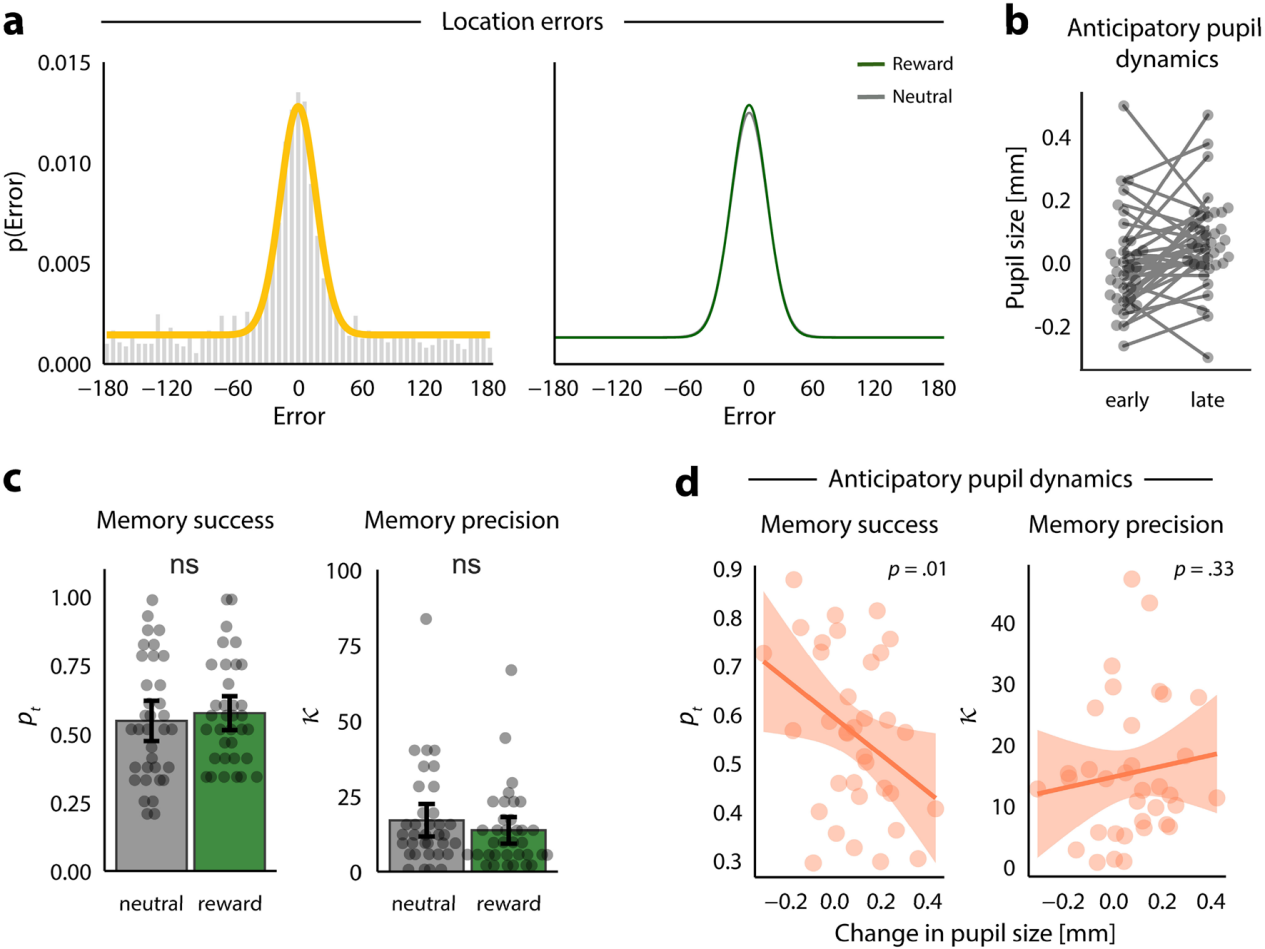
Anticipatory change in pupil size predicts memory success but not memory precision. (**a**) Aggregate location errors (response–target; left) with the best-fitting model probability density function overlaid (yellow), and the best-fitting probability density functions overlaid for each condition separately (right). (**b**) A line plot depicting the general increase in pupil size during the anticipation phase. Grey points refer to average pupil size during early anticipation (first 0.2 s) and late anticipation (final 0.2 s) with individual lines connecting the two points per participant. (**c**) Memory success (${p}_{t}$) and memory precision ($\upkappa$) did not differ between conditions (*p*s > 0.31). Grey points depict individual parameter estimates for each condition; bars indicate condition means; error bars depict 95% confidence intervals at the participant level. (**d**) Smaller anticipatory increase in pupil size (late–early of anticipation period) is associated with better memory success (*p* = 0.01) but is not related to memory precision (*p* = 0.33). Because anticipatory pupil size did not interact with condition, individual measures of pupil size and memory were collapsed across the neutral and reward conditions. Orange line refers to smoothed regression line (y ~ x); shaded areas refer to mean ± SEM.

After verifying that our reward anticipation manipulation was successful, we assessed behavioral performance on the memory task in terms of the probability of successful retrieval and the precision of the retrieved location. To separate these two components, we fit a mixture model^28,32^ to participants’ recall errors, where memory success reflects the proportion of randomly distributed responses and memory precision reflects the variation in precision of successfully retrieved locations (see Fig. 3 for simulated data illustrating these two components). Figure 4a shows the aggregated recall error across all participants for both conditions with the best-fitting overall probability density function (left) and the best-fitting probability density functions for the reward and neutral conditions separately (right). Paired *t*-tests on the individual parameter estimates revealed that the proportion of ‘correct’ trials (${p}_{t}$) did not differ between conditions (*t*(34) = − 0.73, *p* = 0.47, reward: 0.58 ± 0.03, neutral: 0.55 ± 0.04). Similarly, and contrary to our expectations, the precision ($\upkappa$) of successfully retrieved trials was not higher for the reward trials (13.67 ± 2.27) than for the neutral trials (17.02 ± 2.77; *t*(34) = 1.03, *p* = 0.31; Fig. 4c). Moreover, Bayesian analyses revealed moderate evidence in favour of no effect of reward anticipation on both components of memory performance (Bayes factors: ${p}_{t}$= 0.23, $\upkappa$ = 0.3).

#### Pupil and behaviour

First, we explored the relationship between memory performance (memory success and memory precision) and pupil size on the participant-level. We predicted memory success (${p}_{t}$) and memory precision ($\upkappa$) using condition (reward, neutral) and three pupil size regressors: pupil size before (pre-item), during (choice), and following (anticipation) item encoding as well as the interaction between condition and all pupil indices. Interestingly, participants with a larger anticipatory increase in pupil size closely following item encoding (Fig. 4b) showed lower memory success (*t*(34) = 2.7, *p* = 0.01, BF_10_ = 4.4; Fig. 4d). In contrast, average pupil size before and during item encoding was not related to memory success (pre-item main effect: *t*(34) = 1.0, *p* = 0.33, BF_10_ = 0.05; choice main effect: *t*(34) = − 0.84, *p* = 0.41). Nor was there a main effect of condition on memory success (*p* = 0.16) or an interaction effect between condition and pupil size (*p*s > 0.20). Unlike memory success, the average change in anticipatory pupil size did not predict memory precision ($\upkappa$) (*t*(34) = 1.39, *p* = 0.18, BF_10_ = 0.8; Fig. 4d), nor were the other pupil size predictors (pre-item, choice), condition (reward, neutral) or their interactions related to memory precision (*t*s < 1.05, *p*s > 0.30). Importantly, Bayesian analyses revealed substantial (BF_10_ = 0.21) and anecdotal (BF_10_ = 0.97) evidence against an effect of pupil size during choice on subsequent memory success and precision, respectively.

Finally, we examined whether trial-to-trial variability in pupil size might correspond to variability in memory performance. For memory success, we fit a binomial generalized linear mixed-effects model with memory success (binary correct (1) vs. incorrect (0)) as the outcome variable and three pupil size events (pre-item, choice, anticipation) and condition (reward, neutral) as predictors of interest and task block as a predictor of no interest. In line with the participant-level results, items were less often retrieved when the increase in pupil size during the anticipation period was large (*z*(34) = − 2, *p* = 0.046, Bayesian model *b* = − 0.3, 95% CI [− 0.58,  − 0.01]). Pupil size before item onset (pre-item) and during the choice were not associated with memory success (pre-item: *z*(34) = − 0.65, *p* = 0.52, Bayesian model *b* = − 0.04, 95% CI [− 0.27, 0.16]; choice: *z*(34) = 1.1, *p* = 0.27, Bayesian model *b* = 0.2, 95% CI [− 0.09, 0.49]). In addition, there was no effect of condition (*z*(34) = 0.61, *p* = 0.54, Bayesian model *b* = 0.12, 95% CI [− 0.14, 0.3]). To determine whether the *quality* of correctly retrieved items (memory precision) depended on pupil size, we fit a linear mixed-effects model with memory precision (absolute error of correct trials) as the outcome variable and the same predictor variables as the previous model. Memory precision, however, was not associated with the anticipatory increase in pupil size on the trial level (*t*(34) = 0.78, *p* = 0.44), nor with the other pupil-size predictors, or condition (*t*s < 1.39, *p*s > 0.17).

## Discussion

The aim of the current study was to examine how changes in reward-related arousal during memory formation correspond to memory retrieval and variability in the precision of the retrieved memory. Reward was associated with faster reaction times on the number classification question and a larger increase in anticipatory pupil size compared to neutral trials. These findings align with prior research that successfully induced reward anticipation responses, as indicated by decreased reaction times^2,3^ and a stronger pupil size increase^33^ in anticipation of rewards. Nevertheless, contrary to our expectations, we did not observe any memory benefits for items associated with rewards compared to items without any reward association, or a link between pupil size during encoding and subsequent memory. Interestingly, irrespective of reward, a larger change in pupil size in anticipation of the number classification question was negatively predictive of later memory success, though it was not linked to memory precision. Together, these results provide new insights into the effects of reward anticipation on the strength of memory encoding and the role of pupil-linked arousal in the success and quality of remembered events.

We successfully induced a state of reward anticipation in our participants as evidenced by both behavioral and physiological responses. Reaction time on the number classification question (the monetary incentive delay task component) was faster for reward trials than for neutral trials. This finding is in line with previous studies using a similar rewarded reaction time task^2–4,33–35^ and suggests that participants were motivated to receive the reward. We also observed a larger increase in pupil size following stimuli that were predictive of a reward. Schneider and colleagues^33^ were the first to interrogate pupil dynamics during reward anticipation. They reported that participants showed a strong and continuous increase in pupil size in response to a reward cue, which reached its maximum size just before the actual trial outcome. Similar to this, we found a strong reward-related effect when we examined pupil size in the final part of the anticipation phase, just before the target number appeared. Furthermore, both our study and Schneider et al.^33^ found a link between trial-by-trial pupil size and reaction time. Specifically, trials with a larger pupil size response were associated with faster reaction times, suggesting that an increase in arousal just before task engagement boosts subsequent performance. In addition to event-related pupil size, pre-item pupil size has also been related to reaction time on a subsequent task^36^. In the current study, we did not observe any link between trial-by-trial baseline pupil size and reaction time, suggesting that baseline arousal levels did not facilitate subsequent task performance. Together, our findings suggest that pupil size may serve as an indicator of reward anticipation-related increases in arousal and that this change in pupil size can track behavioral performance in subsequent reaction time tasks.

Despite successfully inducing reward anticipation during encoding, we did not observe any significant effect of reward on memory success. There could be several reasons for this. Firstly, it might be that the duration of the consolidation period (i.e., 30-s counting task) was insufficient to reveal the impact of reward on memory^1^. Previous studies that have measured memory success using categorical measures (i.e., ‘old’, ‘new’ responses) have found reward-related memory benefits after a retention period of 24 h or longer^2,3,9,10^, others observed effects after short delays up to 1 h^6–9,36,37^, while some found no effects following short delays^2,38^. This is the first study to assess the effect of reward on continuous measures of memory, for which the memory test is more difficult than for categorical memory tests. In a pilot study we found chance-level performance after a 24-h retention interval, which led us to adopt a short retention interval. Future research should systematically explore reward-related memory effects at different consolidation intervals to pinpoint the minimum time required for memory benefits to take place. Secondly, ours and most other studies have implemented a reward anticipation manipulation at the level of individual trials. This approach assumes that the effects of phasic reward-related dopamine bursts only occur within a short time window (i.e., impact the item only on that trial). However, despite only observing phasic pupillary responses, it is reasonable to speculate that sustained anticipation of reward may lead to a tonic upregulation of SN/VTA activity through prolonged interactions between the hippocampus and the SN/VTA^14,16^. This would result in a ‘spill-over’ effect, whereby memory for neutral items coming after reward items might also be enhanced. Indeed, it has been found that a rewarding context also improves memory for neutral events embedded within it, and that these effects are tightly linked to the recruitment of the hippocampal-SN/VTA system^39^.

We also did not find any reward-related benefits on memory precision. The previously mentioned reasons (e.g., consolidation time and item-specific effects) may contribute to this null finding. However, given that our study is the first to directly investigate this topic, we also cannot rule out the possibility that reward anticipation-induced arousal does not influence memory precision. For example, one related study, to which we can draw parallels, used emotional images to explore the effects of negative, neutral and positive emotions on memory success and memory precision of neutral items^40^. Compared to the neutral condition, memory precision was enhanced following negative emotion induction, but not positive emotion induction, while memory success did not differ between conditions. In the current study, we used a motivational manipulation (e.g., delivering performance-contingent rewards), whereas Xie and Zhang used an emotional manipulation (i.e., directly inducing affectively valenced subjective experience) to induce arousal. Despite using different types of manipulations, both the current study and Xie and Zhang’s study induced arousal during encoding, evidenced by arousal-related effects on subjective ratings^40^ and pupil size and reaction times (in the current study). Importantly, compared to neutral images, Xie and Zhang found enhanced memory precision *only* following negative emotion induction, although the subjective ratings of arousal for positive and negative images were comparable. They suggested that the effect of negative emotion on memory precision was due to valence and not arousal. Indeed, arousal and valence influence memory through distinct processes (see^41^ for review). In the current study, we exclusively manipulated arousal levels and did not address valence, which could explain the absence of reward-related memory precision benefits. Further research is needed to understand whether and under which conditions reward might influence memory precision, possibly taking valence into account.

Unexpectedly, pupil size during item encoding was not associated with later memory success or memory precision. The subsequent memory effect on pupil size is complex in nature^42,43^, with some evidence suggesting an important moderating role for the nature of the encoding task. For example, studies that have employed low-level incidental encoding have found a negative relationship between pupil size during encoding and subsequent memory^44,45^, whereas studies that have used intentional encoding have generally found a positive relationship^46,47^ but see^48^. Several authors have argued that pupil dilation during intentional encoding does not reflect memory formation per se but rather the effortful nature^44^ or the time pressure^49^ that are often associated with intentional encoding tasks. Although we, like others^50^, did not find a subsequent memory effect in either direction, some of these criticisms may also apply to our intentional encoding task; although participants had plenty of time to observe the man-made and natural items, the task required an effortful categorical decision and a manual mouse movement that required some precision. Indeed, the changes in pupil size associated with these mental and motoric processing demands^51,52^, as well as the additional complexity of the monetary incentive delay task and ensuing extra variance in memory performance (see below), may have masked a potential relationship between pupil size during encoding and subsequent memory performance. Therefore, it remains to be seen whether memory precision is related to pupil size during *incidental* encoding, in a simpler task design.

Finally, we found that the change in pupil size during the anticipatory period of the monetary incentive delay component of the study phase was associated with memory success, but not memory precision. Somewhat counterintuitively, this relationship was negative, whereby a larger increase in pupil size was linked to a lower probability of successful retrieval. This might be explained as the result of an attentional shift away from the to-be-remembered item and towards the monetary incentive delay component of the trial. The presence of a larger anticipatory increase in pupil size suggests that on those trials, participants were engaging in active preparation for the upcoming number classification task. In accordance with interference theory^53^, this shifting of attention towards the target number might have interfered with the early stages of memory consolidation of the previously presented item, at a moment when the memory trace was most vulnerable^54^. In other words, we hypothesize that dual-task inference caused a trade-off between performance on the two tasks the participant was asked to perform in close succession; and that trial-to-trial variability in the amount of trade-off caused the negative correlation. In conclusion, our study aimed to investigate the relationship between reward-related arousal during memory formation and memory retrieval accuracy and precision. While reward anticipation led to faster reaction times and increased pupil size responses, no significant memory benefits were observed for reward-associated items. In addition, pupil size before and during encoding was not related to later memory performance. Notably, regardless of reward, smaller increases in anticipatory pupil size post-item encoding were associated with greater memory retrieval success, possibly reflecting a tradeoff between resources dedicated to item encoding and competing tasks. Further research is needed to fully understand the role of reward in memory precision and the timing of memory-associated pupil responses.

## Method

### Participants

Forty-eight healthy participants completed the study. All participants met the following inclusion criteria: fluent in English, with normal or corrected-to-normal vision and hearing, no history of psychiatric or neurological disorders, no learning disabilities or head traumas. Twelve participants were excluded because they scored at chance level on the memory test (see *Behavioural analysis* for exclusion criteria), leaving a final sample size of 36 (mean age = 23.6 years, range = 19–33 years, women = 25). From this sample, two participants carried out only four of the six blocks, due to time constraints. Three participants were missing one block of behavioural data due to technical issues with the data files, and a further three participants were missing pupil data from one block due to technical issues with the eye-tracker. Participants were told to abstain from using alcohol and caffeine in the 12 and three hours before each session. All participants were paid or received study credits for their participation and provided written informed consent. The study was approved by the Psychology Research Ethics Committee at Leiden University and all methods were performed in accordance with the guidelines given and the regulations overseen by the Ethics Committee.

### Materials

The task was programmed in the Expyriment Python package^55^. The to-be-remembered stimuli were made up of 146 items obtained from a published stimulus set^56^ and by using Google Image Search. Half of the items belonged to the category ‘man-made’ and half of the items to the category ‘natural’. All items were grey-scaled, luminance-corrected to an average RGB value of 125 and resized using custom Matlab and Python scripts. Each of the items was presented in the centre of a grey screen (RGB: 125, 125, 125; Fig. 1). A circle with a radius of 324 pixels surrounded the items. Two coloured dots, one red (RGB: 232, 72, 72) and one green (RGB: 60, 255, 60) signaled a ‘reward location’ and a ‘neutral location’ on the circle. The circle as well as all text stimuli that were presented on each trial (i.e., fixation cross, number, and feedback) were equal in luminance to the background (RGB: 60, 60, 255). Pupil diameter was measured at a sample rate of 40 Hz using a Tobii Pro X3-120 eye-tracker (Tobii, Danderyd, Sweden). We used a chinrest to ensure that the eye-tracker was positioned approximately 75 cm from the participant’s eyes, and to restrict head movements during the task. The experiment was carried out under constant ambient light. Before the experiment began, the eye-tracker was calibrated using a default five-point calibration method from the eye-tracker manufacturer.

### Design and procedure

The location memory paradigm was made up of 84 trials divided into six study-test blocks. In the study phase of each block (Fig. 1), a trial began with a fixation screen (duration 3–6 s, uniformly distributed), followed by the presentation of a man-made or natural item in the centre of the circle. Half of the participants were informed that they could obtain a reward following a picture of a man-made item (e.g., chair, shoe, car), the other half were informed that they could obtain a reward following a picture of a natural item (e.g., fruit, bird, hand). The item order was randomised for each participant. After three seconds, a green and a red dot appeared on the circle, indicating the reward location and neutral location on that trial. The angles of these locations were varied across trials, with the constraint that the two dots were always spaced 180 degrees apart. To this end the circle was segmented into steps of four degrees, and each of these angles (e.g., 8, 12, 16, 20°) appeared once in a randomised order. Participants were instructed to answer the question ‘Do you expect a reward on this trial?’ by moving the item to the correct location. If the item belonged to the reward category, the correct location was centered on the green dot. If the item belonged to the neutral category, the correct location was centered on the red dot. Participants used the mouse to drag the item to one of the locations and clicked the left mouse button to finalise their item location response. If participants placed the item greater than 20 degrees away from the correct dot, the response was considered incorrect. Participants had three seconds to make their response. After three seconds or a mouse click response, the item remained on screen for a further three seconds. Participants were encouraged to respond correctly to this part of the trial, since a reward was only possible given a correct item classification. Participants were instructed to pay attention to each item’s specific location and were told they would undergo a memory test after each block.

The second part of the trial started with the presentation of a fixation screen (duration 2–4 s, uniformly distributed). Next, participants carried out a number classification task^2,57^: a target number (1, 4, 6, or 9, randomised) was presented for 100 ms and participants responded to the question: ‘Is the number higher or lower than 5?’, by pressing the up or down arrow as quickly as possible using the middle or index finger of the left hand. The trial ended with an empty screen (1 s), followed by the presentation of a feedback stimulus for 0.5 s. On reward trials (i.e., item belonging to the reward category), participants either received positive feedback (+15c) if they responded correctly and before a given response deadline, or received negative feedback (−10c) if they responded incorrectly or too slowly. The response deadline was individually titrated based on the participant’s reaction times in the preceding trials so as to yield positive feedback on ~ 70% of the reward trials. On neutral trials (i.e., item belonging to the other category), the feedback was always neutral (0c). At the end of the study phase (14 trials), the total reward earned on that block was presented on the screen. At the end of the experimental session, participants received the total reward money earned on all blocks.

To prevent rehearsal of the previously studied items, before the test phase there was a 30-s delay during which participants counted backwards from a randomly generated number in steps of 3. During the test phase of each block, participants were presented with the 14 items they had just studied, as well as eight lures, in a randomised order. First, they indicated whether or not they had studied the item in the preceding study phase as well as their confidence in this response. Then a circle appeared around the item and participants were instructed to recall the location on the circle that was associated with that item during the study phase. Once participants were ready to respond, they used the mouse to move the item to that location on the circle and clicked the left mouse button to finalise their choice. Participants were given 10 s to respond to each item. Responses to the eight lures were not analysed. Prior to the experiment, participants practiced the task for approximately five minutes, during which they could already win and lose money.

### Behavioural analysis

For each trial, we calculated the degree of error (0 ± 180°) between the item location response during the item classification part of the study phase and the item location response during the memory test. Twelve participants who performed at chance level on the memory test were excluded from all analyses. Chance level was defined as a mean absolute error (response–target) of 75° or more^58^. Recall errors were then analysed by fitting a mixture model^28,32^ separately for each condition (reward, neutral) and for each participant. This model has been shown to characterise long-term memory performance in visual long-term memory tasks^20,24,40,58–60^ similar to the present study. The mixture model assumes that two distinct sources of error contribute to participants’ retrieval performance across trials: (1) the presence of random guesses (i.e., failed retrieval responses), which is modelled by a uniform distribution; and (2) the variability (or noise) in successful retrieval of the target location, which is modeled by a circular Gaussian (i.e., von Mises) distribution centered around a mean error of 0°. After removing incorrectly classified trials (‘Do you expect a reward on this trial?’), one participant had too few remaining trials for model fitting. Therefore, the mixture model was applied to the data of 35 participants. Using maximum likelihood estimation, we fit the two-component mixture model^28^

$$p\left(\widehat{\theta }\right)=\left(1-{p}_{u}\right){\varnothing }_{\upkappa } \left(\widehat{\theta }- \theta \right)+ {p}_{u}\frac{1}{2\pi }$$

where $\widehat{\theta }$ is the participant’s response location (in radians), $\theta$ is the target (encoded) location, ${p}_{u}$ is the proportion of randomly distributed responses, and ${\varnothing }_{\upkappa }$ represents a von Mises distribution with a mean of zero and concentration $\upkappa$. From this, two parameters were estimated: ${p}_{t}$, which reflects the probability of successful retrieval (computed by subtracting ${p}_{u}$ from 1); and $\upkappa$, a measure of dispersion, with higher values reflecting more precise memory representations. These parameters were estimated at the group level (aggregated across all trials and per condition) and at the participant level (per condition).

Because the number of trials available for fitting the mixture model (84 per participant) was relatively small compared to similar previous studies (e.g., Cooper and Ritchey^58^; Richter et al.^24^), our model estimates may have been somewhat noisy. To verify that our results were not driven by potentially aberrant model estimates, we carried out an outlier detection analysis on the condition-wise model estimates, whereby any individual with an estimate greater than 2.5 standard deviations away from the condition mean was considered an outlier. Two participants with exceptionally high precision (Fig. 4c) fell outside this threshold for memory precision ($\upkappa$). When we repeated all analyses involving memory performance after removing these two participants, all significant effects remained and no new significant effects were found.

To interrogate memory performance on the trial level, we quantified trial-specific measures of memory success (correct or incorrect) and memory precision^58^. To do this, we used the mixture model probability density function that provided the best fit of the aggregate location errors (Fig. 4a). Trials were considered correct (incorrect) if the location error was more (less) likely under the von Mises distribution than under the uniform distribution that together formed the mixture distribution. This resulted in a threshold of ± 33 degrees. Trial-level memory precision was defined for the correct trials as the inverse absolute location error, such that higher values reflect higher precision.

### Pupil data preprocessing and analysis

Pupil data was preprocessed using PupCor (https://github.com/lindvoo/PupCor) and pupil preprocessing and analyses were implemented in Python 3, Matlab R2021a and RStudio. The location memory paradigm we used required participants to move their gaze during a trial. Evidence suggests that gaze movements introduce a confound in the pupil size measurements, since the eye rotates away from the camera^61,62^. To correct for this as much as possible, we started with applying pupil foreshortening error correction to the pupil data. This was carried out using PsPM [version 6.0.0], available at http://bachlab.org/pspm in accordance with the methods proposed by Hayes and Petrov^61^. After applying this correction for each participant, we analysed the pupil (left or right) for which more data points were available. Blinks were automatically marked by the device manufacturer and were removed by applying an automated interpolation procedure from 100 ms before blink onset to 400 ms after blink offset. The data were then manually checked and corrected if any artifacts had not been successfully removed. The pupil timeseries was then low-pass filtered using a 10-Hz fourth-order Butterworth filter with zero phase shift.

After preprocessing, the pupil data were segmented into three events of interest: pre-item (0.2 s before item onset), choice (0 s to 0.2 s after red/green dots appeared, i.e., choice display), and anticipationꟷa change measure that we defined as the difference in pupil size between the final 0.2 s and the first 0.2 s of the anticipation period (duration 2–4 s, uniformly distributed). With the choice event, we aimed to capture the pupil response associated with item encoding. To account for the sluggish response of the pupil and still capture potential reward-related arousal effects, we chose a short event window at the onset of the choice display, since this also allowed us to minimize the confounding effect of hand movement on the pupil (i.e., when participants begin to move the item into the chosen location). Events in which > 50% of the pupil samples were marked as invalid were excluded from analysis. Pupil size samples that were > 3 SDs outside the event mean were considered spurious and removed from the event epoch. After these criteria were applied, an average of 90 ± 2% trials remained for the pre-item event, 92 ± 2% for the choice event, and 86 ± 3% for the anticipation event. An inspection into the pre-item event revealed that there were carry-over effects from the pupil response on the previous trial. Specifically, if the previous trial was a reward trial, pre-item pupil size on the subsequent trial was larger than if the previous trial was neutral (*p* = 0.023 [paired *t-*test]). To ensure that these carry-over effects were not confounding the pupil results, we carried out control analyses where the effects of the previous trial condition and ITI duration (3 s–6 s) were modelled as additional predictors of no interest. Finally, the pupil data associated with the choice event were baseline-corrected on the trial level by subtracting the pre-item pupil size. The three events were then averaged across trials, separately for each condition (reward, neutral) and participant. Only correctly classified trials (‘Do you expect a reward on this trial?’) were used for all pupil analyses.

### Statistical analyses

Statistical analyses were carried out using Python 3 and R (version 4.0.3). To estimate the potential effects of reward on memory success, memory precision and pupil dilation, we carried out two-tailed paired-samples *t*-tests using the function *scipy.stats.ttest_rel* (using α ≤ 0.05). To test for the effects of pupil size on memory performance, we used linear mixed-effects models to predict memory success (${p}_{t}$) or memory precision ($\upkappa$) using three pupil size events (pre-item, choice, anticipation) using functions *lmer* and *glmer* (libraries *lmerTest* and *lme4* in R). To account for individual deviations from fixed group effects, intercepts were modeled as random effects in the participant-level models and intercepts and slopes were modeled as random effects in the trial-level models. To account for possible time-on-task effects, task block (1 to 6) was always included as a predictor of no interest in all trial-level models. In addition, to quantify the evidence for any effects of interest, we carried out Bayesian paired-samples *t*-tests (library *BayesFactor* in R) and Bayesian linear mixed-effects models (library *rstanarm*, functions: *stan_lmer* and *stan_glmer* in R). We report Bayes factors, except for all trial-level models where we report Bayesian model estimates and confidence intervals (mean [95% confidence intervals]). We used the default Cauchy prior *r* = 0.707. Throughout the paper, data are expressed as mean ± SEM unless otherwise mentioned.

## Data Availability

The processed data as well as code to reproduce the results are publicly available without restriction: https://github.com/bethlloyd/Lloyd_Nieuwenhuis_MemPrecision.
